# Retroperitoneal cystic lymphangioma coexisting with a uterine fibroid in a 42-year-old woman: A case report

**DOI:** 10.1016/j.crwh.2024.e00646

**Published:** 2024-08-23

**Authors:** Fatima El Hassouni, Sofia Mchichou, Samia Sassi, Najat Lamalmi, Samir Bargach, Mounia Yousfi malki, Siham El Haddad, Kenza Berrada

**Affiliations:** a Mohammed V University of Rabat, Rabat, Morocco; b Mohammed V Souissi University

**Keywords:** Retroperitoneal lymphangiomas, Cystic lymphangiomas, Retroperitoneal masses, Case report, Congenital lymphatic channel, Surgical excision

## Abstract

Lymphangiomas are rare benign neoplasms traditionally thought to result from congenital lymphatic channel malformations, though they may also be associated with other conditions. Retroperitoneal lymphangiomas account for 1% of all lymphangiomas, and fewer than 200 cases have been reported. A 42-year-old woman was admitted with symptoms of abdominal pain and distension. A computerized tomography (CT) scan showed an abdomino-pelvic mass and a giant uterine myoma. The patient underwent explorative laparotomy and the whole cyst mass was removed along with the uterine myoma. Cystic lymphangiomas are often misdiagnosed because of the vague symptoms and the absence of obvious etiology. A provisional diagnosis can be made with CT but histological examination confirms the diagnosis. Cystic lymphangioma should be included in the differential diagnosis of an ovarian cystic mass. Complete resection can be curative.

## Highlights

• Adult retroperitoneal cystic lymphangiomas are rare benign lymphatic neoplasms.
• The presenting symptoms are generally non-specific.
• Retroperitoneal cystic lymphangioma is diagnosed by histopathological evaluation following cystectomy.
• Complete surgical resection is the optimal treatment for retroperitoneal cystic lymphangioma.

## 1 Introduction

Lymphangioma is a rare benign neoplasm that was initially described by Koch in 1913. It stems from a congenital malformation of the lymphatic channels, brought about by an obstruction in the lymphatic ducts, resulting in lymphangiectasia [1]. The tumor is infrequent, but its precise incidence remains unknown. The retroperitoneal cystic lymphangioma predominantly manifests in the pediatric age group, with approximately 90% of cases diagnosed before the age of 2 [1]. Lymphangioma in intraabdominal sites (in particular retroperitoneal sites) are extremely uncommon, especially among adults. Retroperitoneal lymphangiomas represent only 1% of all lymphangiomas [2].

## 2 Case Presentation

A 42-year-old nulligravida woman presented to the gynecology ward with complaints of abdominal pain and distension. She had no medical or surgical history. Physical examination revealed an enlarged uterus extending to the umbilicus.

Ultrasound **showed** a coarsely oval latero-uterine cystic mass molding the surrounding structures, with an echogenic structure containing cystic portions and some thin septas. Pelvic magnetic resonance imaging (MRI) showed a large abdomino-pelvic mass in the retroperitoneal site **that was fused through**.

**Fig. 1:**
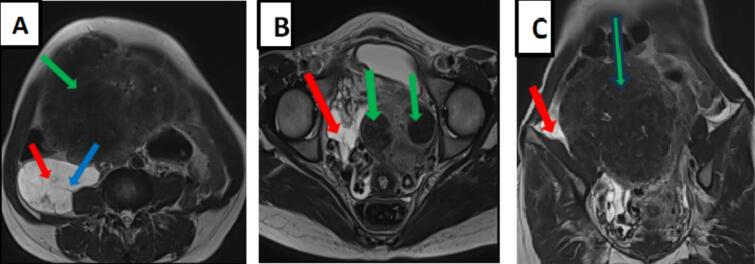
Axial sections (A,B) and a coronal section (C) on T2-weighted pelvic MRI showing an abdominopelvic cystic (red arrow) molding adjacent structures containing thin septa (blue arrows) associated with a polymyomatous uterus in T2 hyposignal (green arrow). (For interpretation of the references to colour in this figure legend, the reader is referred to the web version of this article.)

**Fig. 2:**
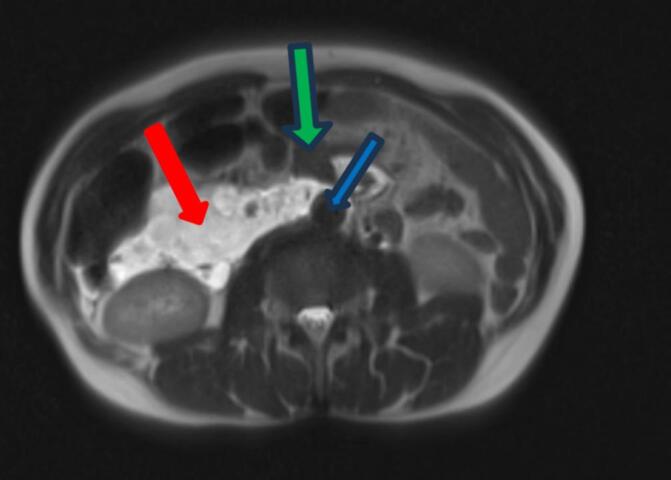
T2-weighted axial section at the top of the renal pedicle on pelvic MRI. The extension of the mass (red arrow) is in intimate contact with the aorta (blue arrow) as well as the head of the pancreas (green arrow). (For interpretation of the references to colour in this figure legend, the reader is referred to the web version of this article.)

**Fig. 3:**
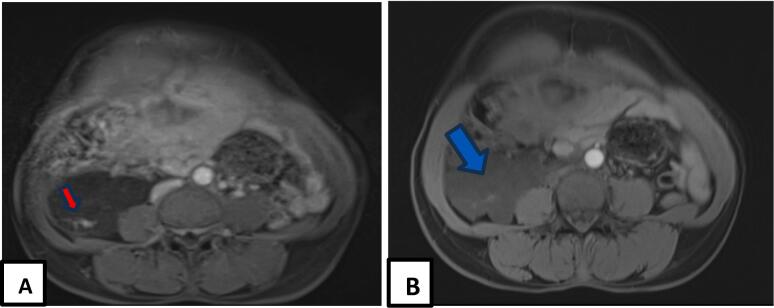
Axial sections on T1-weighted pelvic MRI without (A) and with (B) contrast injection showing the septa with a discrete T1 hypersignal (red arrow) and that the mass moderately increased after injection of the contrast medium (blue arrow). (For interpretation of the references to colour in this figure legend, the reader is referred to the web version of this article.)

The adjacent structures, extending to the level of the right kidney, encompassed the inferior cava vein, coming into contact with aorta and the posterior surface of the head of the pancreas. This mass had a well-defined contour, heterogeneous signal intensity in T2, isosignal intensity in T1, enclosing thin septa, without restriction to diffusion, and moderately enhancing after injection of the contrast agent (Fig. 1, Fig. 2, Fig. 3). Additionally, the uterus was found to be polymyomatous with multiple myomas classified as FIGO 5 and 6. MRI findings pointed to the presence of a right abdomino-pelvic mass suggestive of peritoneal inclusion cyst with a polymyomatous uterus.

**Fig. 4:**
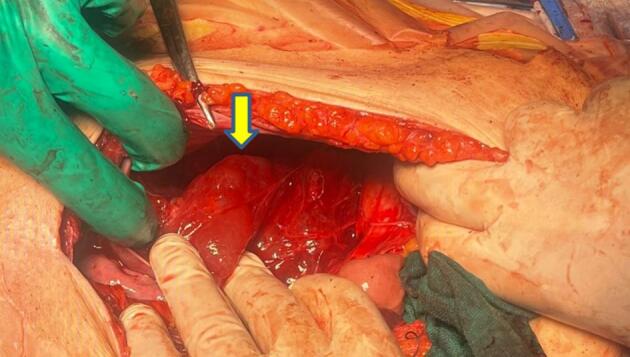
Intraoperative image after opening the peritoneum revealing the cystic mass (yellow arrow). (For interpretation of the references to colour in this figure legend, the reader is referred to the web version of this article.)

Intraoperatively, a large uterine fibroid was noted and after opening the peritoneum the cystic mass (Fig. 4) was found between the right kidney and the pancreas. It was completely resected after meticulous isolation from its surroundings. A myomectomy was also performed. The patient recovered without any complications.

**Fig. 5:**
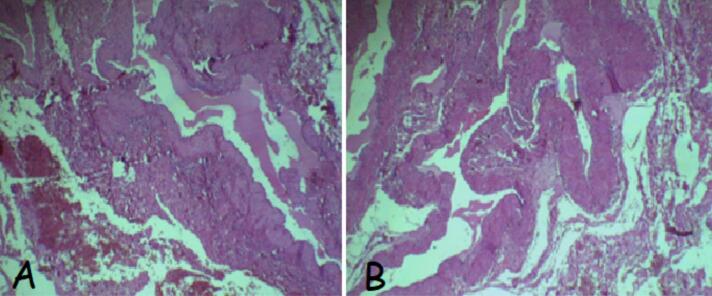
A and B: Histopathological findings: Dilated lymphatic channels are lined by uniform endothelial cells without atypia, surrounded by focal areas of fibrosis with scattered lymphocytes (HE, Gx 20).

For pathological examination a specimen was fixed with 10% buffered formalin, with hematoxylin-eosin staining for light microscopy (Fig. 5). The first fragment showed a fusocellular proliferation of intersecting bundles of smooth muscle cells. These cells had regular elongated nuclei with rounded tips and abundant eosinophilic cytoplasm. There was no mitotic activity. The mass was a uterine leiomyoma measuring 25 × 19 × 20 cm and weighing >1 kg. The second fragment measured 9x3cm and weighed 30,9 g. It had dilated and anastomosing thin-walled blood vessels, lined by a single layer of flat endothelial cells without atypia. The stroma supporting the cystic spaces was fibroblastic, with lymphocyte aggregates and small, variable-sized congestive vessels. This confirmed the diagnosis of lymphangioma.

## 3 Discussion

Lymphangioma is an uncommon benign neoplasm that originates from abnormal development of the lymphatic channels. Most lymphangiomas are diagnosed in childhood and their presentation in adults is rare [3]. The usual sites of cystic lymphangiomas are head, neck (75%) and the axillary area (20%). In the case reported here, the location was retroperitoneal, which is very rare, representing only 5% of cystic lymphangiomas [4].

Some theories suggest that lymphangiomas arise either from a congenital malformation of lymphatic vessels, or from other causes, especially in adults (encompassing mechanical pressure, abdominal trauma, radiotherapy, fibrosis or inflammation) [1,3,5]. The etiology in the present case is uncertain. There were no identifiable precipitating factors such as trauma, infection, or radiation exposure, and there was no history of prior surgery.

Some studies have found a statistically significant association between the diagnosis of cystic lymphangioma on MRI and various quantitative features of uterine leiomyomas, such as the number of fibroids, the maximum diameter of the largest lesion, and the total volume of the leiomyomatous uterus. These findings suggest that an enlarged leiomyomatous uterus might disrupt lymphatic circulation, potentially leading to the formation of cystic lymphangioma. The larger the uterine fibroid, the higher is the risk of cystic lymphangioma [6].

Cystic lymphangiomas are commonly asymptomatic and found incidentally during gynecological examination for a different clinical complaint or during surgery [3]. Unfortunately, there are no pathognomonic signs or symptoms of retroperitoneal lymphangiomas. Commonly presenting symptoms include: abdominal distension, abdominal pain, and asymmetry due to the enlarging mass [[4], [5], [6], [7]]. The patient reported here presented with abdominal pain and distension, similar to retroperitoneal lymphangiomas. However, these symptoms can also be related to leiomyoma feature.

Imaging plays a crucial role in diagnosis. An ultrasound scan is the first-line examination because of its non-invasiveness and wide availability but it is often challenging to assess the retroperitoneal region with this method [1]. Cystic lymphangiomas usually appear as a well-limited unilocular or multilocular fluid tumor, in which the cysts are separated by thin partitions. However, the contents of cysts, which are often **anechoic**, can become echogenic during intracystic hemorrhage or contain some calcifications [8].

CT allows for better determination of the density of these tumors and for better evaluation of their relation with the neighboring organs, leading to improved preoperative assessments and more precise procedures. On CT scan, cystic lymphangioma typically presents as a large, multi-septated cystic mass with thin walls. The attenuation values of the cysts can vary, from that of liquid to that of fat. They often have an elongated shape, while calcification of the cyst wall is rare [9].

On MRI, cystic lymphangiomas present a diverse appearance, influenced by their internal contents, which can be chylous, serous, hemorrhagic, or mixed. Typically, lymphangiomas appear hypointense or isointense to muscle on T1-weighted images and hyperintense on T2-weighted images. This characteristic pattern aids in their identification and differentiation from surrounding tissues [10]. Unfortunately, these different imaging modalities are insufficient to establish an accurate diagnosis; they help only to determine size, location, presence of invasion, and characteristics of contents [5].

The differential diagnosis of a retroperitoneal cyst includes: abscesses, ovarian cysts, pancreatic pseudocysts, sarcomas, a cystic teratoma, and mucinous pancreatic neoplasms or microcystic pancreatic adenoma [3,5]. However, other benign and non-aggressive congenital or low-malignant cystic masses may be indistinguishable from retroperitoneal lymphangioma but are rare or less common [9]. The pre-operative diagnosis is rare and histopathological examination of the surgical specimen remains necessary for definitive diagnosis [1].

Histologically, the diagnosis of a lymphangioma is based on specific criteria, encompassing the presence of a cystic lesion with or without an endothelial lining, a distinct and well-defined wall containing clusters of lymphoid tissue, and a stroma composed of collagen and fibrous tissue [3]. Double staining with Prox1 and CD31 is the most reliable method for characterizing lymphangioma endothelial cells. The pre-operative diagnosis is rare and histopathological examination of the surgical specimen remains necessary for definitive diagnosis [1].

Total (rather than partial) cystectomy is the optimal treatment for retroperitoneal lymphangiomas as it prevents recurrence and avoids the mass extending into adjacent retroperitoneal compartments, displacing organs and vessels. It can compress and infiltrate vital structures or cause complications like intracystic hemorrhage, cyst rupture, volvulus, or infection. In case of suspicion of organ invasion or when other organs too close, the extent of the procedure can be extended, such as bowel resection [10]. In some rare circumstances, patients may need non-surgical therapeutic options including US/CT-guided aspiration and injection of sclerosant agents [5,11,12].

The coexistence of uterine fibroids and retroperitoneal lymphangioma in a 42-year-old nulliparous woman can significantly complicate fertility. Uterine fibroids may impair fertility by distorting the uterine cavity and increasing miscarriage risk, while retroperitoneal lymphangiomas, though rare, may indirectly affect reproductive organs if large enough. Treatment options, particularly surgical interventions, may further impact fertility, making a multidisciplinary approach essential [13,14].

After surgical resection, the appropriate follow-up plan for patients is still uncertain [1]. Although the literature lacks in-depth studies on the diagnosis of retroperitoneal cystic masses, it is crucial to differentiate neoplastic lesions from non-neoplastic ones.

## 4 Conclusion

Adult cystic retroperitoneal lymphangioma is a rare tumor which is generally diagnosed at the pathologic examination. It results from a congenital malformation of the lymphatic channels, though it can also be associated with other factors. Cystic retroperitoneal lymphangioma is a challenge for physicians due to its infrequency and non-specific clinical symptoms, and further studies are crucial to identify diagnostic tests that aid in the early identification of these tumors before surgery. Typically, confirmation of the diagnosis requires surgical removal followed by histopathological examination.
